# *Human Herpesvirus 6*-Associated Hemophagocytic Syndrome in a Healthy Adult

**DOI:** 10.3201/eid0801.010149

**Published:** 2002-01

**Authors:** Hiroshi Tanaka, Tetsuo Nishimura, Mikiko Hakui, Hisashi Sugimoto, Keiko Tanaka-Taya, Koichi Yamanishi

**Affiliations:** Osaka University Medical School, Osaka, Japan

**Keywords:** *Human herpesvirus 6*, virus-associated hemophagocytic syndrome, G-CSF

## Abstract

Virus-associated hemophagocytic syndrome is a fulminant disorder associated with systemic viral infection and characterized pathologically by multiple-organ infiltration of hemophagocytic histiocytes into the lymphoreticular tissues. This is the first report of a previously healthy adult in whom *Human* *herpesvirus 6* reactivation induced this syndrome with severe hemodynamic and respiratory distress.

Virus-associated hemophagocytic syndrome (VAHS) is a fulminant disorder associated with systemic viral infection and characterized pathologically by multiple-organ infiltration of hemophagocytic histiocytes into the lymphoreticular tissues. VAHS has been associated with *Epstein-Barr virus* (EBV), cytomegalovirus (CMV), adenovirus, and *Herpes simplex virus* (HSV), as well as with a variety of nonviral infections (*1*). There have been several reports of VAHS in children caused by *Human herpesvirus* *6* (HHV-6) infection. We report what may be the first record of reactivated HHV-6 causing VAHS with severe hemodynamic and respiratory distress in a previously healthy adult.

## Case Report

A healthy 22-year-old man with a high fever lost consciousness and was admitted to our hospital. On admission, a skin rash covered his whole body; cervical, axial, inguinal, and supraclavicular lymphadenopathy and hepatosplenomegaly were observed. The leukocyte count was 14,590/mm^3^, the hemoglobin concentration 13.9 g/dL, and platelet count 12.7x10^4^/mm^3^. Elevated liver enzymes (glutamate oxalacetic transaminase 155 U/L, glutamate pyruvate transaminase 379 U/L) were found, along with elevated lactate dehydrogenase 911 U/L (normal 130-290 U/L) and C-reactive protein 4.9 mg/dL (normal <0.2 mg/dL). Serum antibody tests for CMV and HSV were negative, but a serum antibody test for EBV was positive (1:640) on day 2 after admission. Infectious mononucleosis was suspected, and the case was managed conservatively without antibiotics for 13 days after admission.

**Figure 1:**
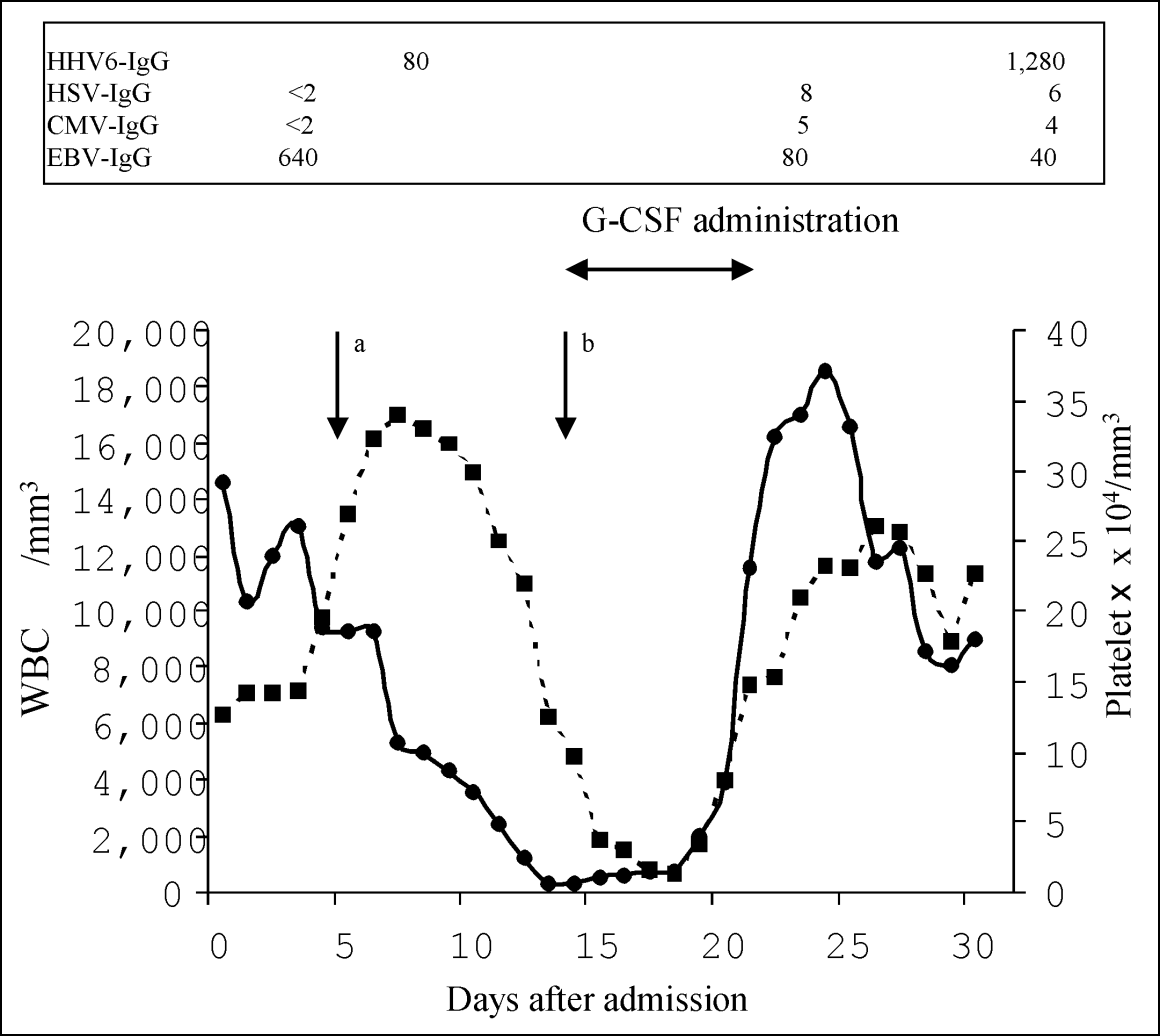
Changes in leukocyte count (solid circles), platelet count (solid squares), and serum antibodies tests for *Human herpesvirus 6* (HHV-6), *Herpes simplex virus* (HSV), cytomegalovirus (CMV), and *Epstein-Barr virus* (EBV) after admission. Values in the box demonstrate immunoreactivity to HHV6, HSV, CMV, and EBV. ^a^HHV-6B was isolated from peripheral blood mononuclear cells on day 5 after admission. ^b^Pancytopenia was diagnosed on the day 14 after admission. IgG = immunoglobulin G; G-CSF = granulocyte-colony stimulating factor (G-CSF).

**Figure 2:**
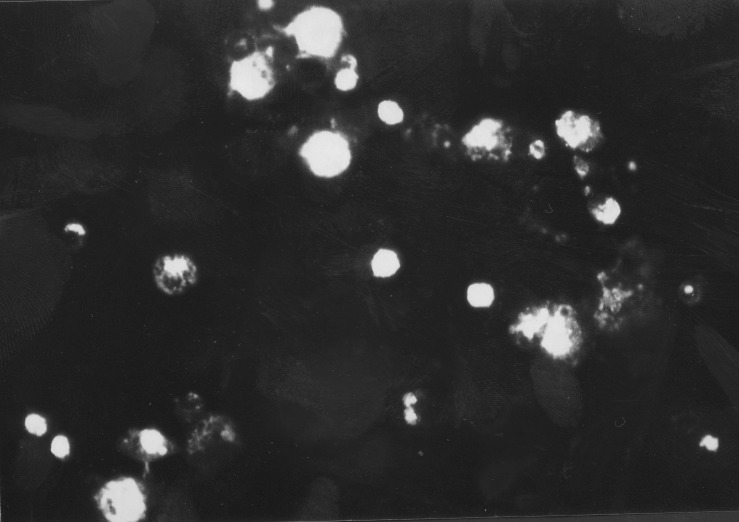
Immunofluorescence micrograph of peripheral blood mononuclear cells infected with *Human herpesvirus 6B* isolated on day 5 after admission.

On day 14, the patient suddenly went into shock and severe respiratory distress developed, with PaO_2_ 45 mmHg and PaCO_2_ 35 mmHg at FiO_2_ of 100%. Pancytopenia was evident, with a leukocyte count of 270/mm^3^, hemoglobin of 9.1 g/dL, and platelet count of 9.7x10^4^/mm^3^ (Figure 1). The patient’s bone marrow was hypocellular, with a nucleated cell count of 1.6x10^4^/mm^3^ (normal 13.7-23.1x10^4^/mm^3^), and showed an increased number of histiocytes with hemophagocytosis and mature large granulolymphocytes. Elevated serum concentrations of tumor necrosis factor (TNF)-alpha (44 pg/mL; normal <15.6 pg/mL), interleukin (IL)-1² (129 pg/mL; normal <3.9 pg/mL), IL-6 (3,415 pg/mL; normal <3.1 pg/mL), IL-8 (15,598 pg/mL; normal <31.2 pg/mL), and granulocyte-colony stimulating factor (G-CSF) (165,000 pg/mL; normal <39.1 pg/mL) were observed. Blood and organ bacteria cultures were negative. The CD4/CD8 ratio (0.73; normal 0.88-1.84) was low, and complete suppression of immunoglobulin was observed, with decreased immunoglobulin (Ig) A (30 mg/dL; normal 115-440 mg/dL), IgG (620 mg/dL; normal 1,000-2,060 mg/dL), and CD19 (1.1%; normal 9.7-17.3 %). Serum antibody tests for HHV-6 were positive (1:80 on day 7 and 1:280 on day 30 after admission), suggesting that this was a case of HHV-6 reactivation. HHV-6B was isolated as previously described (*2*) from peripheral blood mononuclear cells (PBMC) on day 5 after admission (Figure 2), suggesting VAHS induced by HHV-6.

On day 14 after admission, at the onset of pancytopenia, subcutaneous administration of recombinant human G-CSF (Lenograstim; Chugai Pharmaceutical Co. LTD., Tokyo, Japan) was started at 2 µg/kg. Improvement in hematologic parameters was apparent 8 days after the start of G-CSF, and there were no further complications (Figure 1). With mechanical ventilation and fluid resuscitation with catecholamine, the respiratory and hemodynamic status improved. HHV-6B was not isolated from PBMC on day 32 after admission. The symptoms and signs of VAHS disappeared completely, and the patient was discharged 44 days after admission.

## Conclusions

VAHS is characterized by prominent phagocytosis of erythrocytes and nucleated blood cells in the bone marrow and lymph nodes. The general symptoms are fever and hepatosplenomegaly. Some cases have been been associated with hypercytokinemia by TNF-alpha, IL-1-beta, and interferon (IFN-gamma), resulting in severe hemodynamic collapse and acute lung injury (*3*). Lymphocyte activation induces excessive production of IFN-gamma, which acts on a variety of cells, resulting in macrophage activation and tissue damage. In keeping with this proposed injury mechanism, successful treatment with cyclosporin A has been documented (*3*). We administered G-CSF alone, although the serum G-CSF concentration was markedly increased when VAHS was diagnosed, suggesting remarkable up-regulation. HHV-6 is a lymphotropic virus that grows in PBMC. It is widespread in the normal population; >80% of the general population in Japan is seropositive. Exanthema subitum has been considered a manifestation of primary infection with HHV-6 (*2*). There have been several reports of VAHS in children caused by HHV-6 infection (*4*,*5*). The few adults who escape HHV-6 infection during childhood and acquire primary HHV-6 infection as young adults have a self-limited, febrile illness, usually associated with lymphadenopathy and resembling infectious mononucleosis (*6*). To our knowledge, this is the first report of a healthy adult in whom HHV-6 reactivation induced VAHS with severe hemodynamic and respiratory distress.
